# ‘Does the National Antibiotic Guideline- 2008 remain applicable for treating diabetic foot infection?’ A new evidence-based regional study on culture and sensitivity patterns in Terengganu population

**DOI:** 10.5704/MOJ.1403.017

**Published:** 2014-03

**Authors:** SD Balakrishnan, NJ Shahid, TM Fairuz, IMA Ramdhan

**Affiliations:** Department of Orthopaedics, Hospital Sultanah Nur Zahirah, Kuala Terengganu, Malaysia; Department of Orthopaedics, Hospital Sultanah Nur Zahirah, Kuala Terengganu, Malaysia; Department of Orthopaedics, Hospital Sultanah Nur Zahirah, Kuala Terengganu, Malaysia; Department of Orthopaedics, Hospital Sultanah Nur Zahirah, Kuala Terengganu, Malaysia

## Abstract

Diabetic foot infections make up a significant number of orthopaedic ward admissions. The recommended choice of empirical antibiotics used in Malaysia for diabetic foot infections is based on the National Antibiotic Guidelines 2008. The pattern of bacteriology and the effectiveness of the treatment of diabetic foot infections based on this guideline were analyzed through a retrospective study in our hospital. Data over a period of one year (May 2012- April 2013) was analyzed, and 96 patients were included in this study. Polymicrobial growth (58%) was mainly isolated, followed with an almost equal percentage of gram-negative (22%) and gram-positive organisms (20%). The empirical antibiotics based on the national antibiotic guidelines were used as definitive antibiotics in 85% of the cases. Although there was slight variation in the pattern of organisms as compared to other studies conducted in this country, the high rate of positive clinical response proved that the antibiotic guideline was still effective in diabetic foot infection treatment.

**Key Words:**

Diabetic foot infections, National Antibiotic Guidelines, Culture and Sensitivity

## Introduction

Diabetic foot complications, especially infections such as wet gangrene, infected ulcers, abscess and necrotizing fasciitis are - the leading causes of non-traumatic amputations in Malaysia ^1^. Apart from surgical intervention, the appropriate choice of antibiotics is an important part in the management of these wounds. In Malaysia, we have been using the National Antibiotic Guideline that was published in 2008 as a main guideline for the empirical antibiotic choice for diabetic foot infections. Up to date, there are no recent studies done in our country regarding microorganism growth pattern and its response to the treatment as recommended by this Guideline. Thus, it is important to evaluate the efficacy of this guideline as it has been adhered to for about five years now. Therefore, the objective of this study was to look into patterns of bacteriology among diabetic foot patients in our hospital with the focus to identify the commonly isolated microorganisms from diabetic foot infection and to assess the response of the patients to the antibiotics as per the National Antibiotic Guidelines. This is important because, with time, the spectrum of organisms involved in the locality may change and the empirical choice of antibiotic treatment for these patients may not be applicable as recommended. By doing this, we attempt to prevent the misuse of antibiotics which can cause emergence of multi-resistant organisms and superinfection ^2^.

## Materials and Methods

### 

A retrospective study was conducted using the data from the Diabetic Foot Registry of HSNZ, from May 2012 till April 2013. The data comprised of all in-patient diabetic foot patients that have been admitted for diabetic foot related infections with a first positive intraoperative tissue culture (e.g. deep tissues, curetted bone). As proposed by Citron et al, post debridement specimens were obtained, as it would help a better yield of positive cultures ^3^. The samples were transported using sterile bottles to the microbiology lab and cultured using Mueller-Hinton agar. Our exclusion criterion was, diabetic patients with foot infections that have been managed in the in non-orthopaedic wards. Thus out of 182 patients over that one-year period, only 96 fulfilled our criteria. This data was counter-checked using the Hospital Information System (HIS).

Patients were categorized into mild, moderate and severe infection based on clinical features described in the national antibiotic guidelines 2008 ^4^ which was originally adapted from the Infectious Disease Society of America (IDSA) PEDIS classification ^5^, and the antibiotics were started as per protocol for each group (Table I). The response to the treatment was observed based on clinical improvement and the sensitivity to the antibiotics started. The empirical antibiotics started based on the national antibiotic guidelines were changed if there was no positive clinical response or if the organism was resistant to the particular antibiotic chosen.

## Results

A total of 96 patients were evaluated, 68 males (70%) and 28 females (30%). Most of the patients who presented (40%) were diagnosed with diabetes only five years previously -, while 31% and 17% patients had been diagnosed previously with diabetes ranging from 5-10 years and 10-15 years respectively. Only 12% of them had been diagnosed more than 15 years ago. (Table II). The majority of the organisms isolated were polymicrobials (58%), followed by an almost equal percentage of gram negative organisms (22%) and gram positive organisms (20%) (Figure 1). Among the monomicrobials isolated, the majority was Staphylococcus aureus ( 27%), followed by Klebsiella ( 22%) and Streptococcus spp( 15%). (Table III). Clinically, most of the patients (86%; n=83) had moderate infections, majority of which did not require antibiotic change from the empirical therapy that was started. The mild and severe cases, which made up of four and ten patients respectively, responded successfully to the empirical antibiotics that were started (Figure 2). Thus, the overall percentage of patients who were continued on the empirical antibiotics as definitive therapy was 85%.

## Discussion

Our demographic data showed that the vast majority of the patients were males. This may be due to the fact that in Terengganu most males in the lower socioeconomic groups are manual labourers or fishermen and tend to have a higher incidence of foot injuries, which is an important risk factor for diabetic foot infections. The diabetic foot infections were also most common in the group diagnosed less than five years ago. This is possibly due to late diagnosis of diabetes mellitus or poor foot care knowledge in this population.

The data accumulated in this study was compared to other recent studies. We compared the bacteriology pattern of diabetic foot infection in our hospital with a recent study from India by Girish et al. They reported predominance of polymicrobial growth, and among the monomicrobials, majority was gram negative organisms ^6^. Our country showed that monomicrobial infection was more common. Nadeem et al in a study 2005 in a teaching hospital our country, reported a predominant gram negative growth ^7^. In 2006, Yoga et al from another hospital in the state of Kedah found that gram positive organisms were more common ^2^. These different patterns of isolates possibly depend on the severity of the infection and geographical variations 5. Based on our observation, patients in our hospital tended to present late for treatment of diabetic foot infections, as they usually sought traditional healers initially. This may be one of the contributing factors to the variation in the isolate patterns. Another contributing factor to the organism types isolated maybe the fact that the patients in this study were mainly those with moderate to severe infections that required inpatient treatment ^3^.

The choice of empirical antibiotics started in our centre was based upon the National Antibiotic Guideline 2008. Once the patients were started on the empirical antibiotics, they were assessed for clinical response and the antibiotic sensitivity of the culture taken intra-operatively was traced. As our results show, 85 % of patients responded to the antibiotics chosen initially and only 15% needed a change in antibiotics due to resistance of organisms or unfavourable clinical response. This positive outcome was seen because the antibiotic coverage of moderate to severe infections was relatively broad spectrum and was important especially in-patients who were immunocompromised.

Early recognition of the severity of infection, medical stabilization, appropriate antibiotic selection, early surgical intervention and plans for delayed reconstruction are crucial components of managing diabetic foot infections. The empirical antibiotic therapy is started for coverage of pathogens, while patient is being stabilized metabolically and hemodynamically, and while timing surgical intervention, when appropriate. Once the patient is medically stabilized, initial surgical debridement is performed with the goal of resecting all non-viable tissue and decompressing gross abscess. This is of utmost importance for successful control of the infection^8^.

In recent times, the role of biofilm has been studied as a cause of chronic diabetic foot wounds. Studies have well documented the presence of biofilm as an important barrier to effective treatment. A study by James et al showed that 60% of chronic wounds contained biofilm as compared to only 6% of acute wounds9. With this new knowledge related to diabetic foot wounds, we should include synergy of different antibiotics for better biofilm penetration and eradication in a revision of the National Antibiotic Guidelines of this country.

**Table I: Summarized National Antibiotic Guideline 2008 [4]:**
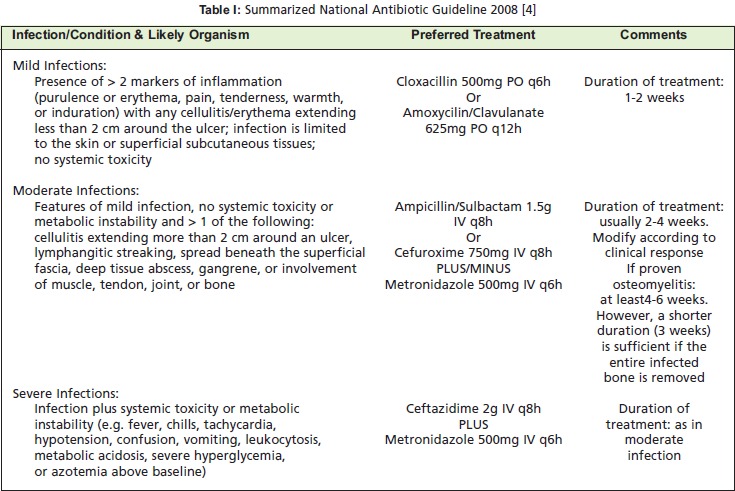


**Table II: Duration of diabetes:**
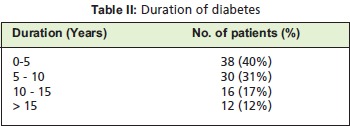


**Table III: Spectrum of monomicrobials isolated:**
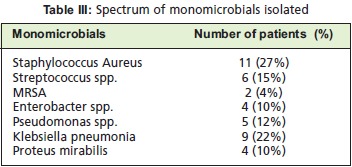


**Figure I: Overall pattern of microbials isolated.**
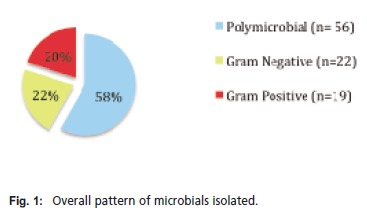


**Figure II: The response of patients to treatment based on National Antibiotic Guidelines 2008.**
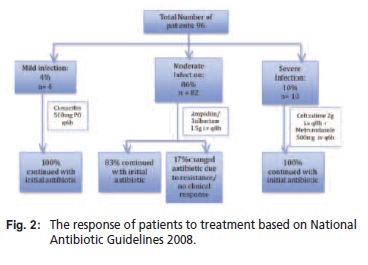


## Conclusion

The culture and sensitivity pattern of the microorganisms isolated in our hospital is consistent with studies done in other centres, and the empirical antibiotic therapy as recommended by the National Antibiotic Guideline 2008 of Ministry of Health of Malaysia is effective in the treatment of diabetic foot infections. We suggested some modification to increase effectiveness against biofilm producing organisms in future editions. Based on this study, apart from surgical intervention, the proper identification of causative organisms and the appropriate antibiotic therapy of diabetic foot infections are key contributors to the achievement of successful outcome.

## References

[1] Yusof MI, Sulaiman AR, Muslim DA (2007). Diabetic foot complications: a two-year review of limb amputations in a Kelantanese
population. Singapore Med J.

[2] Yoga R, Khairul A, Sunita K, Suresh C (2006). Bacteriology of diabetic foot lesions. Med J Mal.

[3] Citron DM, JC Goldstein EJC, Merriam CV, Lipsky BA, Abramson MA (2007). Bacteriology of Moderate-to-Severe Diabetic
Infections and in vitro Activity of Antimicrobial Agents.. J Clin Microbiol.

[4] (2008). National Antibiotic Guideline, 2008 – Ministry of Health Malaysia. http://hsbas.moh.gov.my/v2/uploads/nag.pdf.

[5] Lipsky BA, Berendt AR, Cornia PB, Pile JC, Peters EJ, Armstrong DG (2014). 2012 Infectious Diseases Society of America
Clinical Practice Guideline for the Diagnosis and Treatment of Diabetic Foot Infections.. http://www.idsociety.org/uploadedFiles/IDSA/Guidelines-Patient_Care/PDF_Library/2012%20Diabetic%20Foot%
20Infections%20Guideline.pdf..

[6] Bergdoerker GN, Kumar TN (2011). Culture & Sensitivity pattern of microorganisms isolated from diabetic foot infection in a tertiary
hospital.. Int J Cur Biomed Phar Res.

[7] Nadeem SR (2007). Microbiology of diabetic foot infections in a teaching hospital in Malaysia: a retrospective study of 194 cases.. J Microbiol Immunol Infect.

[8] Claire MC, John JS (2010). Diabetic foot infections: a team-orientated review of medical and surgical management. Diabetic Foot & Ankle.

[9] James GA, Swogger E, Wolcott R, Pulcini Ed, Secor P, Sestrich J (2008). Biofilms in chronic wounds.. Wound Repair Regen.

